# Remote Symptom Alerts and Patient-Reported Outcomes (PROS) in Real-World Breast Cancer Practice: Innovative Data to Derive Symptom Burden and Quality of Life

**DOI:** 10.3390/bioengineering11080846

**Published:** 2024-08-19

**Authors:** Emelly Rusli, Debra Wujcik, Aaron Galaznik

**Affiliations:** Carevive by HealthCatalyst, Boston, MA 02110, USA; debra.wujcik@gmail.com (D.W.); aaron.galaznik@healthcatalyst.com (A.G.)

**Keywords:** breast cancer, patient-reported outcomes, remote symptom monitoring, quality of life, breast cancer treatment, symptom alerts, real-world evidence

## Abstract

Treatment for breast cancer (BC) can lead to debilitating symptoms that can reduce outcomes and quality of life (QoL). Symptom surveillance using a remote symptom monitoring (RSM) platform enables the capture and reporting of patient-reported outcomes (PROs) from home. Women with BC used an RSM platform to complete weekly surveys and report any symptoms experienced during treatment. Symptoms reported as moderate/severe generated alerts to the clinical team. Clinical actions in response to the alert were captured. Results highlighted the value of data generated from a PRO-generated alert system to characterize longitudinal symptom burden and QoL in real-world BC practice, particularly in patients with poor functional status. The most prevalent symptoms that resulted in alerts were pain, nausea/vomiting, neuropathy, fatigue, and constipation. Most women reported one or more moderate/severe symptoms that generated an alert with an average of two alerts per week. Patients with frail status had more alerts, worse QoL and higher treatment bother, indicating that frail patients may benefit from continuous monitoring of symptoms, function, and QoL over time. A case study of patients without pre-existing peripheral neuropathy showed the rapid trajectory from the first report of mild neuropathy until alerts were generated, making a case for early intervention.

## 1. Introduction

Women undergoing breast cancer (BC) treatment may experience debilitating symptoms, such as peripheral neuropathy and pain that can significantly reduce treatment outcomes and quality-of-life (QoL) [1]. Prior studies have shown the value of routine symptom monitoring and patient-reported outcomes (PROs) assessments on oncology patient outcomes including reduced symptom burden, decreased healthcare resource utilization, and improved survival [2,3,4]. 

Continued surveillance using a remote symptom monitoring (RSM) platform enables capture of PROs from home using devices such as smart phones, tablets, and personal computers [2]. Unlike the conventional follow-up process where symptoms and patients’ concerns are not addressed until the next clinical visit or are immediately escalated to a higher level of care, the RSM system allows sending PRO results directly to the electronic medical record (EMR) to inform the healthcare team and support monitoring of patient status [2]. PRO monitoring is especially useful for patients identified as high risk for side effects, such as those who are frail and/or have comorbidities [5]. 

The most useful RSM platforms include an alert system to flag the healthcare team when symptoms worsen and require clinical intervention prior to the next clinic visit [6]. An alerting system employed in patients with poor functional status allows the clinical team to monitor treatment response and make treatment adjustments in real time. Insights on patient experiences with symptom prevalence and their impact on daily function and QoL, particularly in patients with poor functional status, are essential for treatment decision making [4,5,6]. 

Chemotherapy-induced peripheral neuropathy (CIPN) is an example of a symptom experienced by women undergoing treatment for BC that can be particularly debilitating. The overall prevalence of CIPN ranges from 19% to over 85% [7]. In BC adjuvant treatment, 42% of women report numbness and tingling after two years, with 10% stating the symptoms are severe [7]. Patients with moderate-to-severe CIPN have significantly impaired tactile, vibratory, and thermal thresholds compared to patients without CIPN [8]. Severe CIPN interferes with daily function, reduces quality-of-life, and may lead to treatment interruption or dose reduction [8]. Risk factors for CIPN include age, pre-existing neuropathy (PX PN), and treatment with taxanes and platinum-based drugs [9].

This research highlights the use of data collected from an RSM platform with a PRO-generated alert system to characterize symptom burden and QoL by frailty status, describe the clinical actions implemented by the healthcare team in response to reported symptom alerts, and illustrate a case study of using the data to explore the trajectory of neuropathy in real-world BC practice.

## 2. Materials and Methods

### 2.1. Patient Population and PROmpt^®^

Study participants included women with BC who consented and enrolled in Carevive PROmpt^®^, an RSM platform, between September 2020 and November 2023 (study period). Patients were enrolled by their care provider during a clinic visit as part of the standard of care. Patients consented to the end user license agreement (EULA) and were informed that their de-identified data would be added to the secure database registry and used for other purposes such as research. Since the requirements of informed consent documentation were met, a waiver of consent was awarded by the institutional review board (IRB). Patients completed the baseline survey, which captured demographics, clinical characteristics, and frailty status. Patients then received weekly surveys to report any symptoms experienced during treatment. Sixteen symptoms (anxiety, constipation, cough, diarrhea, decreased appetite, fatigue, general pain, insomnia, mouth/throat sores, musculoskeletal pain, nausea, neuropathy, rash, sadness, shortness of breath, and vomiting) were derived from PRO-CTCAE^®^ [10] and asked in the weekly surveys. Patients had the opportunity to report QoL (derived from the QoL/global health items of EORTC QLQ-C30) [11], physical function (measured by PROMIS 4A physical function) [12], and the degree of treatment bother (measured with a single item FACT-GP5) [13] in the same survey. A PRO-CTCAE^®^ composite score using reported symptom frequency, severity, and/or interference was calculated in the system and classified symptoms as mild, moderate, or severe [14]. Mild symptom reports generated an education plan sent to the patient and were discussed with the patient at the next clinical visit. Moderate or severe symptom reports triggered an alert to the care team for immediate evaluation and mitigation. The healthcare team reviewed the alert, interacted with the patient, and recorded the response using a drop-down list of actions in the platform. Patient-reported QoL, physical function, and treatment bother data were visible to the healthcare team upon alert generation. Patients were followed from the baseline survey completion to the last completed survey, death, or the end of study period (whichever was earliest).

### 2.2. Symptom Prevalence and Alerts

Baseline demographics and clinical characteristics were described for all patients in the cohort and those who reported at least 1 moderate or severe symptom during the study period. One moderate or severe symptom report would generate one unique alert to the healthcare team. The prevalence of symptoms that triggered an alert was characterized. Symptom burden, defined as the number of alerts per patient per week, was explored by breast cancer stage (early stage: 0–IIIA or late stage: IIIB–IV), biomarker (hormone receptor (HR) positive/human epidermoid receptor 2 (HER2) negative (HR+/HER2−), HER2+/HR+, or triple negative BC (TNBC)), age, baseline frailty status (frail, intermediate, or fit), and Eastern Cooperative Oncology Group (ECOG) performance status (0, 1, or 2+). QoL, physical function, and the degree of treatment bother over 12 weeks, which was the common duration of oncology regimen, were reported by frailty status. Baseline frailty status was assessed using the Carevive modified geriatric assessment (mGA) [15,16], cancer and aging resilience evaluation geriatric assessment (CARE GA) [17,18,19], and patient-reported functional status (PRFS) [20,21]. Clinical actions documented were described, and further free-text exploration of clinical notes were conducted to characterize the actions implemented. A single alert could have multiple clinical actions.

### 2.3. Trajectory of Neuropathy

Further analysis was conducted to explore the neuropathy trajectory in patients who did not have pre-existing neuropathy (PX PN) at baseline, examining time to first neuropathy report and time to alert trigger due to severity. Baseline demographics and clinical characteristics were described. Age at first neuropathy, duration of therapy, cancer stage, race, CIPN drugs use, and the level of neuropathy interference were explored by the difference in time to neuropathy alert trigger. 

### 2.4. Statistical Analysis

This was a descriptive study, i.e., not designed to test the statistical significance of a hypothesis; hence, no priori power calculations were performed, and no statistical comparison was conducted. Baseline demographics, patient characteristics, and clinical actions documented were described and summarized. Mean, standard deviation, median, and range were reported for continuous variables. Count and relative frequency were reported for categorical variables. Average score per patient per week for EORTC QLQ-C30 global health/QoL score, PROMIS 4A T-score, and FACT-GP5 score were summarized by averaging the numeric scores reported by patients on a given survey week. Given the small sample size, only the median and interquartile range (IQR) of neuropathy trajectory time were reported. All statistical analyses were conducted using R statistical software version 4.2.3. 

## 3. Results

### 3.1. Demographic and Baseline Characteristics

A total of 646 female patients with BC were included in the cohort, reporting 19,425 symptoms over a median of 12.3 weeks. Median age was 56 (range: 26–84), and 72.1% were White, 20.4% were Black or African American, 61.8% were early stage, 47.2% were HR+/HER2−, and 77.9% were fit (Table 1). About 80.3% of patients (n = 519) reported a moderate/severe symptom at least once, generating 7641 total alerts. Of patients who generated at least one alert, median age was 55 (range: 26–81), and 71.3% were White, 20.6% were Black or African American, 62.6% were early stage, 47.6% were HR+/HER2−, and 75.7% were fit. Chemotherapy, anti-HER2, and mono endocrine therapy were the most common therapies received when patients triggered their first symptom alerts (Table 1).

### 3.2. Symptoms, QoL, and Alerts

**Figure 1:**
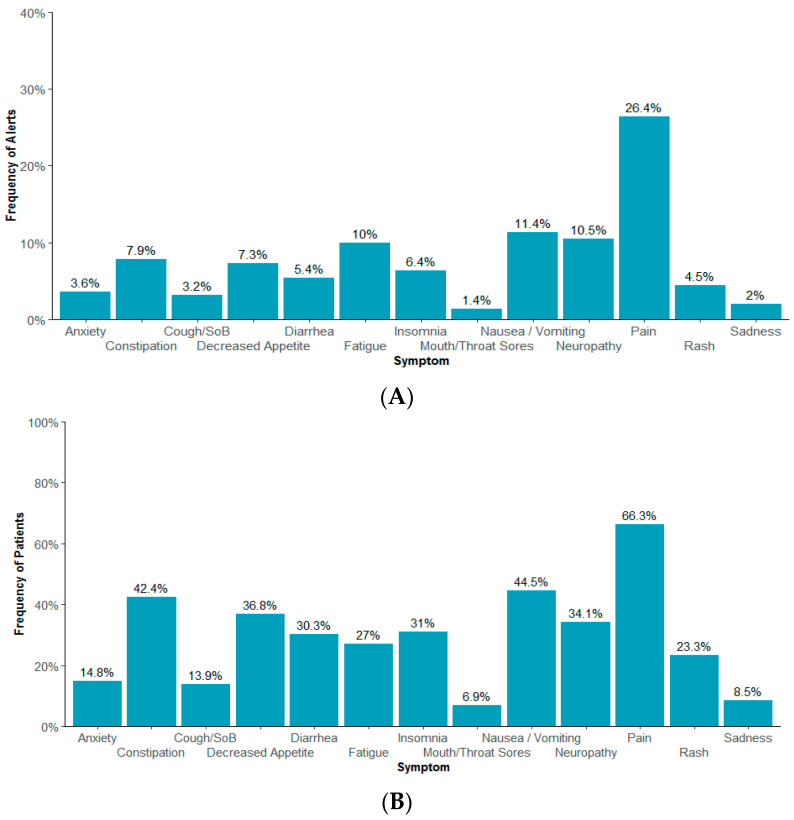
(**A**). Prevalence of alerts resulted from moderate/severe symptoms. (**B**). Prevalence of patients experiencing symptoms that triggered an alert. Cough/SoB = cough or shortness of breath. Nausea/vomiting = nausea or vomiting. There was one unique symptom per alert. Cough or shortness of breath symptom alert would be labeled as ”Cough/Shortness of Breath” to the healthcare team. Similarly, nausea or vomiting symptom alert would be labeled as ”Nausea/Vomiting”.

**Figure 2:**
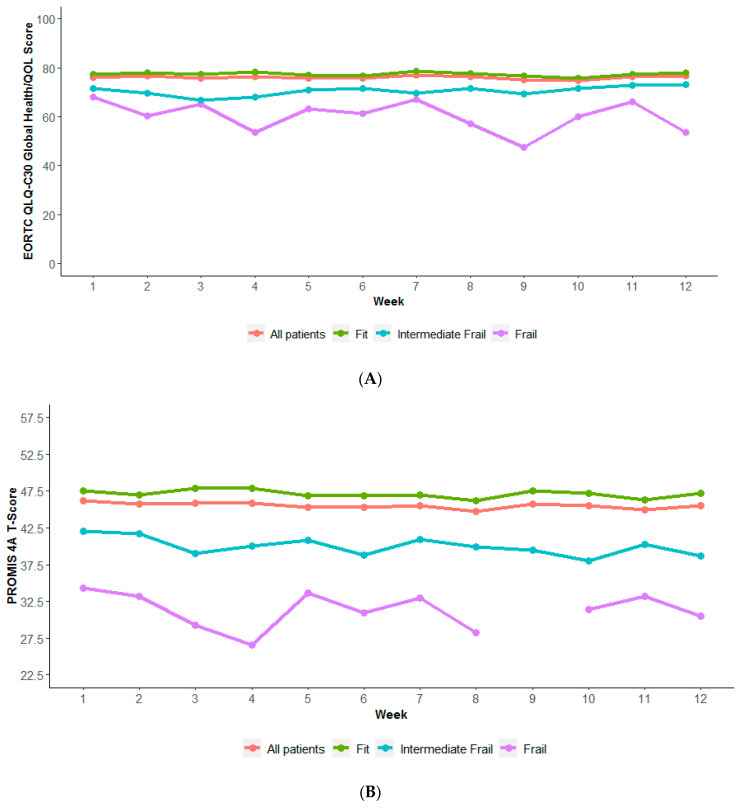

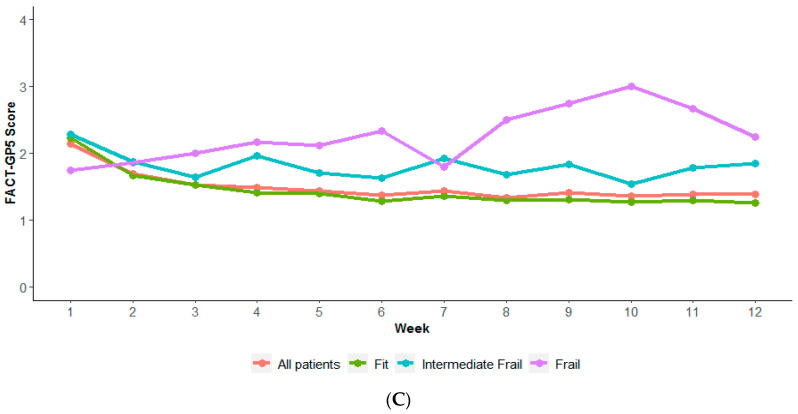
QoL, physical function, and treatment bother by frailty status. (**A**) QoL by frailty status. Average score per patient per week. QoL was measured with global health/QoL items of EORTC QLQ-C30 score (range: 0–100). A higher score indicates a higher, more positive perception of overall health and QoL. (**B**) Physical function by frailty status. Average score per patient per week. Physical function was measured with PROMIS 4A physical function T-score (range: 22.5–57.0). A higher score indicates greater overall function. (**C**) Degree of treatment bother by frailty status. Average score per patient per week. Treatment bother was measured using single item FACT-GP5 (range: 0–4). A higher score indicates a higher degree of treatment bother.

Pain (26.4%), nausea/vomiting (11.4%), neuropathy (10.5%), fatigue (10%), and constipation (7.9%) were the most prevalent symptoms that triggered an alert (Figure 1A). About 66% of *patients* reported pain that triggered an alert at least once, 44.5% of patients reported nausea/vomiting, 42.4% reported constipation, 36.9% reported decreased appetite, and 34.1% reported neuropathy (Figure 1B). Patients generated an average of two alerts (SD = 1.5) per week, with a median of one alert per patient per week (Table 2). The average number of alerts per patient per week was similar by stage, biomarker, and age (Table 2a–c). Frail patients, on average, generated more alerts per week than fit or intermediate patients (Table 2d). A similar trend was observed when comparing ECOG 2+ vs. ECOG 1 or 0 (Table 2d). QoL, physical function, and treatment bother were consistently worse over time for frail than fit or intermediate patients (Figure 2). 

### 3.3. Clinical Actions Documented in the Alerts

**Figure 3:**
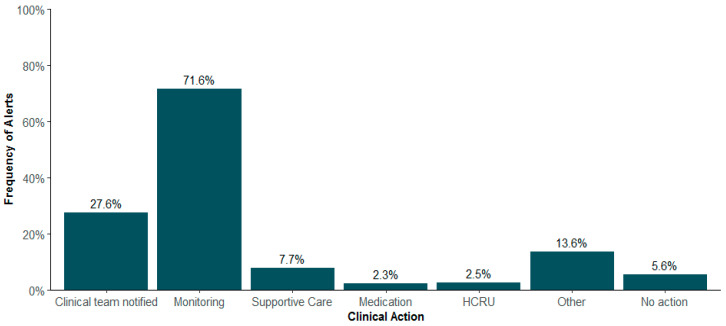
Distribution of clinical actions documented in response to symptom alerts (n = 7641 alerts) HCRU = healthcare resource utilization.

**Figure 4:**
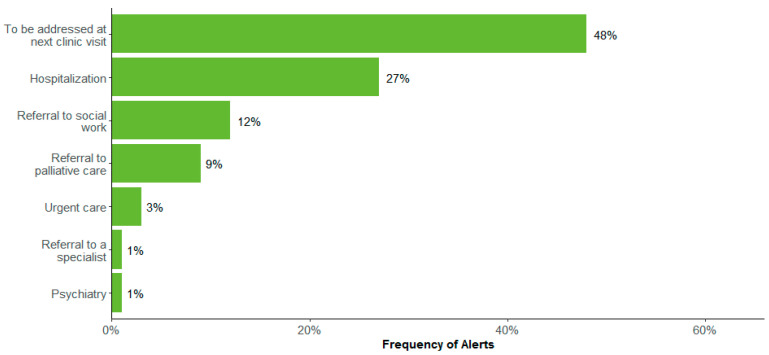
Healthcare resource utilization type (n = 191 alerts).

**Figure 5:**
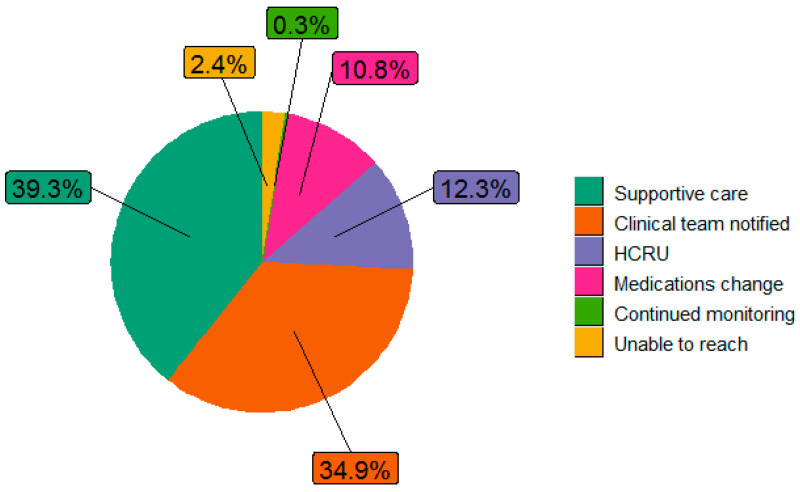
Further exploration of the “Other” clinical action category (n = 1039 alerts). HCRU = healthcare resource utilization. Further HCRU type breakdown: 42% to be addressed at next clinic visit; 15% referral to radiation; 15% other; 13% hospitalization; 10% emergency room; 4% referral to dietitians; 2% referral to social work.

**Figure 6:**
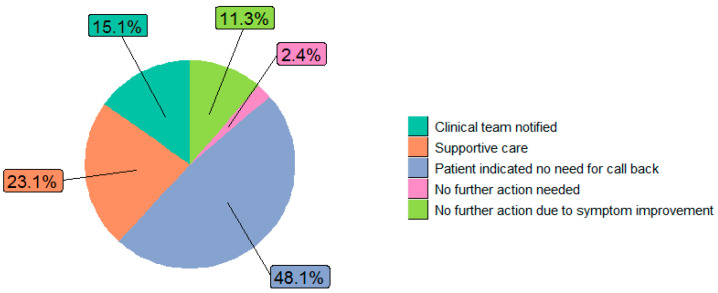
Further exploration of the “No action” clinical action category (n = 212 alerts).

The distribution of clinical actions following the alert were as follows: clinical team notified (27.6%), continued monitoring (71.6%), supportive care (7.7%), medication change (2.3%), and resource utilization (2.5%). “No action” was noted in 5.6% of alerts, and 13.6% of alerts were categorized as “Other” (Figure 3). Almost half (48%) of resource utilization involved addressing the symptom at the next medical appointment, with 27% hospitalization, 12% referral to social work, and less than 10% to palliative care, urgent care, psychiatry, or higher-level care (Figure 4). Further exploration of the “Other” category (n = 1039) showed an additional distribution of clinical actions as supportive care (39.3%), clinical team notified (34.9%), resource utilization (12.3%), medication change (10.8%), unable to reach patients (2.4%), and continued monitoring (0.3%) (Figure 5). Of patients whose alerts were indicated as “No action” (n = 212), 48.1% had indicated no need for a call back, and 11.3% had no further action due to reported improvements in their symptoms (Figure 6). 

### 3.4. Trajectory of Neuropathy

Of the 7641 total alerts generated, 10.5% (n = 801) were associated with neuropathy reported by 177 patients (Table 3). About 58% of patients (n = 103) did not have pre-existing neuropathy (PX PN) and contributed to 56.6% of neuropathy alerts during the study period. Median age at first neuropathy report of patients without PX PN was 56 (range: 28–78), and 58.3% were White, 30.1% were Black or African American, 63.1% were early stage, and 47.6% had prior use of CIPN drugs. Approximately 42% (n = 74) of patients had PX PN, with median age at the first neuropathy report of 58.5 (range: 26–79), and 58.1% were White, 32.4% were Black or African American, 52.7% were early stage, and 60.8% had prior use of CIPN drugs (Table 3).

**Figure 7:**
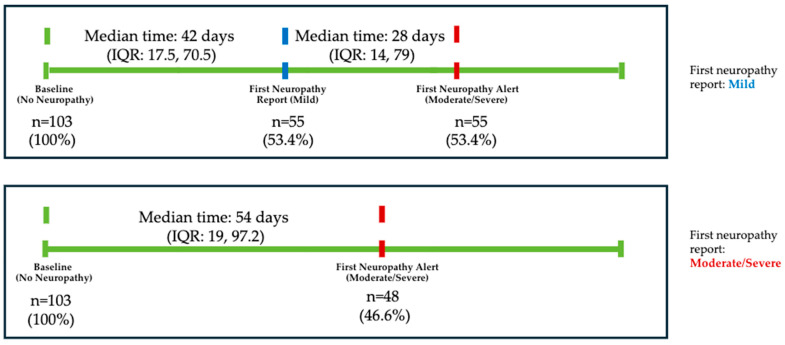
Neuropathy trajectory in patients without pre-existing neuropathy (n = 103).

**Figure 8:**
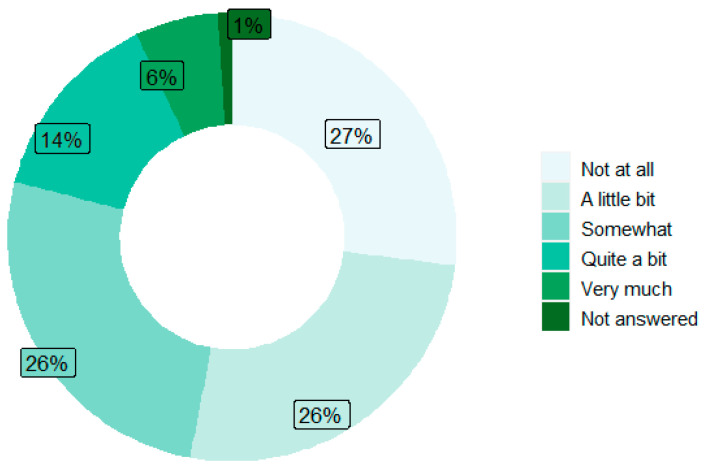
Level of neuropathy interference at first occurrence.

In patients without PX PN (n = 103), neuropathy was first reported over a median of 42 days (IQR: 17.5, 70.5) from baseline (Figure 7). More than half of patients (n = 55) had a mild neuropathy at first report, which then progressed to moderate/severe at a median of 28 days (IQR: 14, 79), Figure 7. About 47% of patients without PX PN reported first neuropathy occurrence as moderate/severe (resulting in an alert) over a median of 54 days (IQR: 19, 97.2) from baseline (Figure 7). The level of interference for first neuropathy occurrence was described to be *A little bit* or *Somewhat* for 52% of patients (Figure 8). Patients with mild first occurrence of neuropathy (n = 55) were then stratified by the time to first neuropathy alert (moderate/severe) from first report: 0–28 days (n = 28) and >28 days (n = 27). For the two groups, median age at first neuropathy was 51 and 49, duration of treatment was 2.4 and 4.4 months, late stage was 17.9% and 14.8%, Black/African American was 32.1% and 22.2%, and receiving CIPN drugs at alert was 50% and 59.3%, respectively. The most prevalent level of interference upon alert was described as *Somewhat* (60.7% vs. 51.9%, respectively), Table 4.

## 4. Discussion

Women undergoing treatment for breast cancer may experience multiple symptoms, some of which resolve with completion of treatment, while others can result in chronic issues [4,22]. To facilitate early intervention before symptoms become severe or permanent, current practice encourages routine symptom monitoring [1,2,3,22]. Electronic systems support monitoring and managing patients at home and between clinical visits. A recent analysis of electronic systems used to report and manage symptoms identified the following desired system features: allows health professional to remotely access and monitor patient-reported data, allows patient to monitor their symptom reports over time, includes a function to send alerts to health professionals for severe symptoms, provides tailored automated patient advice on managing symptoms, provides general patient information about cancer treatment and side effects, includes a feature for patients to communicate with the health care team, and includes a forum for patients to communicate with one another [6]. The Carevive RSM platform provides all of these except for a forum for patients to communicate with one another. 

In this study, all patients reported symptom occurrence throughout treatment, and about 80% had reported one or more moderate/severe symptom reports that created an alert to the healthcare team. An average of two alerts per patient per week indicates the presence of multiple symptoms experienced during treatment. A longitudinal approach to symptom burden provides more insight into the patient experience than a monthly assessment obtained just prior to the next treatment and potentially informs the different interventions needed. Pain was the most prevalent moderate-to-severe symptom reported, present in over 20% of alerts and almost two-thirds of women. Breast cancer pain is multifactorial and requires comprehensive assessment and multidisciplinary interventions [23,24]. Further exploration of the pain etiology, interference with activities of daily living, and response to pain reducing interventions over time in this population is warranted. The etiology and management of the other top symptoms that triggered an alert, nausea/vomiting, neuropathy, fatigue, and constipation, are also multifactorial and deserve further study. A recent analysis of 19 studies that investigated symptom clusters among women undergoing breast cancer treatment showed the most reported clusters were the gastrointestinal cluster (nausea and lack of appetite), pain-fatigue-sleep disturbance and the psychological cluster (anxiety, depression, worry, sadness, nervousness, irritability) [24]. Although this study did not address symptom clusters, further exploration to identify existing symptom cluster changes over time can provide further insight into the patient’s experience. 

Patients with frail status had a poor experience during cancer treatment. Symptom burden measured as number of alerts was higher in frail patients and those with ECOG status 2+. It follows as well that QoL, physical function, and treatment bother were consistently worse for frail than fit or intermediate patients [5]. The baseline assessment of each of these components can help the healthcare team identify patients at higher risk for moderate/severe symptoms and more frequent symptom assessments. 

The value of the RSM is seen in the analysis of clinical actions following the alerts. A robust workflow that allows for prompt notification of moderate/severe symptoms allows real-time response [22]. Studies showed that RSM facilitates engagement between clinicians and patients by giving clinicians the ability to review symptom reports and receive alerts in a timely manner and enabling patients to review their own symptom reports and receive tailored automated advice for symptom management [6,22]. More than 70% required continued/additional monitoring, with 28% requiring specific clinical team notifications. Early intervention can prevent added emergency department visits and hospitalizations [2,3]. Supportive care and medication change provide interventions to address the symptom at home and prevent unplanned clinic appointments or further resource utilization. A small proportion of resource utilization indicates that most symptoms are manageable and that the appropriate level of intervention is given when needed. Multiple clinical actions documented in a single alert facilitates a comprehensive view of the interventions, and the free-text feature within the platform allows the healthcare team to elaborate the actions in nuanced circumstances.

A case study of one symptom, neuropathy, illustrates opportunities for clinical application and multidisciplinary exploration of symptom clusters experienced by breast cancer patients [22,23]. About 34% of the women with at least one alert reported neuropathy, and one in ten alerts was associated with neuropathy. Although 58% of women did not have pre-existing neuropathy at baseline, the same cohort of patients contributed to more than half of the neuropathy-associated alerts, indicating the evolution to moderate-to-severe neuropathy post-baseline. All patients in the study were receiving chemotherapy and/or hormone therapy and may have also had a surgical treatment. Stratification by treatment type may provide further insight into the patient experience [25]. A sub-analysis of neurologic symptom reports in those without PX PN showed the first report of mild neuropathy at a median of 42 days from baseline, with rapid progression to moderate/severe at a median of 28 added days. This finding makes the case that the incidence of neuropathy should trigger an alert to the healthcare team and provide opportunities for earlier intervention to minimize risk of permanent neuropathy [9]. With their capability for obtaining ongoing feedback from patients pre- and post-alert and post-intervention, RSM platforms also represent a potential opportunity for AI (artificial intelligence) applications to both predict symptom worsening and identify optimal interventions.

## 5. Conclusions

RSM is a proven approach to enable the real-time capture of patient symptoms that leads to increased patient satisfaction and improved QoL [2,3,4]. Others have described RSM as a facilitator of clinician and patient engagement by giving clinicians the ability to review symptom reports and receive alerts in a timely manner and enabling patients to review their own symptom reports and receive tailored automated advice for symptom management [6,22]. Data collected from an RSM platform with a PRO-generated alerts system can be used to characterize longitudinal symptom burden and QoL in women with breast cancer undergoing treatment in the real-world clinical practice. Paired with clinical parameters that are collected as part of routine care, (e.g., stage, biomarker, treatment), these data enable rapid characterization of patients and bolster contextualization of PRO results. For instance, early identification of patients with poor functional status, who generated more alerts per patient per week, could allow clinicians to tailor monitoring frequency. As seen in the case study of the peripheral neuropathy trajectory, earlier notification and follow-up of certain symptoms may provide opportunities for earlier intervention and better outcomes. Most alerts generated in this study were addressable with low-intensity interventions, such as enhanced monitoring or clinical team notification, suggesting the importance of timeliness of outreach and response. Lastly, PRO data collected in RSM platforms provide an extraordinary opportunity for increased adoption of predictive modeling and AI towards the future of patient-centered evidence generation in healthcare. Future applications could envision AI applications to leverage direct patient feedback for anticipating clinical events and determining optimal treatment.

## Figures and Tables

**Table 1:** Demographic and baseline characteristics.

	All Patients (n = 646)	Reported at Least 1 Moderate or Severe Symptom (n = 519)
No. of patients with at least one symptom alert, n (%)	519 (80.3)	519 (100)
No. of symptoms reported during study period	19,425	18,506
Total alerts generated during study period	7641	7641
PROs follow-up time (weeks)		
Mean (SD)	19.6 (21.7)	22.3 (22.8)
Median (Range)	12.3 (0–152)	16.1 (0–152)
Age at enrollment (years)		
Mean (SD)	55.6 (12.6)	54.8 (12.7)
Median (Range)	56 (26–84)	55 (26–81)
Age at enrollment (years), n (%)		
<50 years old	200 (31.0)	174 (33.5)
50–64 years old	270 (41.8)	211 (40.7)
65–75 years old	154 (23.8)	118 (22.7)
>75 years old	22 (3.4)	16 (3.1)
Female, n (%)	646 (100)	519 (100)
Race, n (%)		
American Indian or Alaskan Native	7 (1.1)	5 (1.0)
Asian	11 (1.7)	10 (1.9)
Black or African American	132 (20.4)	107 (20.6)
Native Hawaiian or Other Pacific Islander	1 (0.2)	1 (0.2)
White	466 (72.1)	370 (71.3)
Other	9 (1.4)	7 (1.3)
Unspecified	20 (3.1)	19 (3.7)
Biomarker status, n (%)		
HR+/HER2−	305 (47.2)	247 (47.6)
HR+/HER2+	100 (15.5)	85 (16.4)
Triple negative	119 (18.4)	90 (17.3)
Unspecified	122 (18.9)	97 (18.7)
Stage, n (%)		
Early stage (0–IIIA)	399 (61.8)	325 (62.6)
Late stage (IIIB–IV)	147 (22.8)	114 (22.0)
Unspecified	100 (15.4)	80 (15.4)
Baseline frailty status, n (%)		
Fit	503 (77.9)	393 (75.7)
Intermediate	77 (11.9)	69 (13.3)
Frail	44 (6.8)	41 (7.9)
Unspecified	22 (3.4)	16 (3.1)
ECOG status, n (%)		
0	144 (22.2)	120 (23.1)
1	134 (20.7)	108 (20.8)
2+	61 (9.4)	58 (11.2)
Unspecified	307 (47.7)	233 (44.9)
Treatment closest to first symptom alert, n (%)		
Chemotherapy	164 (25.4)	164 (31.6)
Anti-HER2 therapy	101 (15.6)	101 (19.5)
Mono Endocrine therapy (ET)	91 (14.1)	91 (17.5)
PD-1/L1 inhibitors	45 (7.0)	45 (8.7)
CDK 4/6 inhibitors	36 (5.6)	36 (6.9)
Other	33 (5.1)	33 (6.4)
Did not generate alerts	127 (19.7)	0 (0.0)
Unspecified	49 (7.6)	49 (9.4)

**Table 2:** No. of alerts per patient per week by clinical characteristic. (a) No. of alerts per patient per week by stage. (b) No. of alerts per patient per seek by biomarker. (c) No. of alerts per patient per week by age at enrollment (years). (d) No. of alerts per patient per week by baseline frailty and ECOG status.

**(a)**																
		**All**					**Early Stage** **(0–IIIA)**						**Late Stage** **(IIIB–IV)**			
No. of patients with at least one symptom alert		519					325						114			
Total alerts generated during study period		7641					4439						2088			
No. of alerts per patient per week																
Mean (SD)		2.0 (1.5)					2.1 (1.5)						2.1 (1.6)			
Median (Range)		1.0 (1–16)					1.0 (1–13)						1.0 (1–16)			
**(b)**																
		**All**			**HER2−/HR+**					**HER2+/HR+**					**TNBC**	
No. of patients with at least one symptom alert		519			247					85					90	
Total alerts generated during study period		7641			3671					1245					1403	
No. of alerts per patient per week																
Mean (SD)		2.0 (1.5)			2.0 (1.6)					2.0 (1.5)					2.1 (1.7)	
Median (Range)		1.0 (1–16)			1.0 (1–13)					1.0 (1–12)					2.0 (1–16)	
**(c)**																
		**All**		**<50**				**50–64**			**65–75**			**>75**		
No. of patients with at least one symptom alert		519		174				211			118			16		
Total alerts generated during study period		7641		2547				3398			1520			176		
No. of alerts per patient per week																
Mean (SD)		2.0 (1.5)		2.0 (1.6)				2.1 (1.5)			1.9 (1.4)			1.7 (1.0)		
Median (Range)		1.0 (1–16)		1.0 (1–13)				2.0 (1–16)			1.0 (1–11)			1.0 (1–5)		
**(d)**																
	**All**		**Fit**			**Intermediate**			**Frail**			**ECOG 0**		**ECOG 1**		**ECOG 2+**
No. of patients with at least one symptom alert	519		393			69			41			120		108		58
Total alerts generated during study period	7641		5319			1400			621			1235		1579		1145
No. of alerts per patient per week																
Mean (SD)	2.0 (1.5)		2.0 (1.5)			2.1 (1.4)			2.5 (1.5)			1.5 (0.9)		1.9 (1.1)		2.1 (1.3)
Median (Range)	1.0 (1–16)		1.0 (1–16)			2.0 (1–11)			2.0 (1–10)			1.0 (1–7)		2.0 (1–7)		2.0 (1–9)

**Table 3:** Demographic and clinical characteristics of patients with neuropathy alerts (n = 177).

	All Patients (n = 177)	No PX PN (n = 103)	Had PX PN (n = 74)
Total neuropathy alerts generated, n (%)	801 (100)	453 (56.6)	348 (43.4)
Age at first neuropathy (years)			
Mean (SD)	55.8 (12.8)	55.1 (13.3)	56.7 (12.1)
Median (Range)	57 (26–79)	56 (28–78)	58.5 (26–79)
Female, n (%)	177 (100)	103 (100)	74 (100)
Race, n (%)			
American Indian or Alaskan Native	2 (1.1)	0 (0.0)	2 (2.7)
Asian	4 (2.3)	2 (1.9)	2 (2.7)
Black or African American	55 (31.1)	31 (30.1)	24 (32.4)
Native Hawaiian or Other Pacific Islander	1 (0.6)	1 (1.0)	0 (0.0)
White	103 (58.2)	60 (58.3)	43 (58.1)
Other	5 (2.8)	4 (3.9)	1 (1.4)
Unspecified	7 (4.0)	5 (4.9)	2 (2.7)
Biomarker status, n (%)			
HR+/HER2−	76 (42.9)	46 (44.7)	30 (40.5)
HR+/HER2+	31 (17.5)	15 (14.6)	16 (21.6)
Triple negative	35 (19.8)	21 (20.4)	14 (18.9)
Unspecified	35 (19.8)	21 (20.4)	14 (18.9)
Stage, n (%)			
Early stage (0–IIIA)	104 (58.8)	65 (63.1)	39 (52.7)
Late stage (IIIB–IV)	45 (25.4)	22 (21.4)	23 (31.1)
Unspecified	28 (15.8)	16 (15.5)	12 (16.2)
Prior use of CIPN drugs, n (%)	94 (53.1)	49 (47.6)	45 (60.8)

**Table 4:** Characterization of patients whose first neuropathy report was mild, based on the time to first neuropathy alert (n = 55).

	0–28 Days (n = 28)	>28 Days (n = 27)
Age at first neuropathy, years		
Mean (SD)	52.3 (12.5)	52.4 (14.4)
Median (Range)	51 (31–78)	49 (28–77)
Late stage (IIIB–IV), n (%)	5 (17.9)	4 (14.8)
Race, n (%)		
American Indian or Alaskan Native	0 (0.0)	0 (0.0)
Asian	0 (0.0)	1 (3.7)
Black or African American	9 (32.1)	6 (22.2)
Native Hawaiian or Other Pacific Islander	0 (0.0)	0 (0.0)
White	15 (53.6)	18 (66.7)
Other	3 (10.7)	0 (0.0)
Unspecified	1 (3.6)	2 (7.4)
Time since diagnosis (months)		
Mean (SD)	11.6 (24.9)	18.4 (29.2)
Median (Range)	5.7 (1, 134)	5.7 (2.6, 120)
Median duration of treatment (months)		
Mean (SD)	7.0 (18.6)	18.9 (34.8)
Median (Range)	2.4 (0–98.9)	4.4 (1.3–128)
CIPN drugs use at alert, n (%)	14 (50.0)	16 (59.3)
No. of neuropathy alerts per patient/week		
Mean (SD)	1.0 (0.1)	1.0 (0.2)
Median (Range)	1.0 (1–2)	1.0 (1–2)
Level of neuropathy interference at first alert, n (%)		
Not at all	1 (3.6)	0 (0.0)
A little bit	2 (7.1)	2 (7.4)
Somewhat	17 (60.7)	14 (51.9)
Quite a bit	7 (25.0)	4 (14.8)
Very much	0 (0.0)	4 (14.8)
Not answered	1 (3.6)	3 (11.1)

## Data Availability

The datasets presented in this article are not readily available because of privacy restrictions and data usage limitations. Requests to access the datasets should be directed to the corresponding author.

## References

[1] Hamer J., McDonald R., Zhang L., Verma S., Leahey A., Ecclestone C., Bedard G., Pulenzas N., Bhatia A., Chow R. (2017). Quality of life (QOL) and symptom burden (SB) in patients with breast cancer. Support. Care Cancer.

[2] Basch E., Deal A.M., Kris M.G., Scher H.I., Hudis C.A., Sabbatini P., Rogak L., Bennett A.V., Dueck A.C., Atkinson T.M. (2016). Symptom monitoring with patient-reported outcomes during routine cancer treatment: A randomized controlled trial. J. Clin. Oncol..

[3] Basch E., Deal A.M., Dueck A.C., Scher H.I., Kris M.G., Hudis C., Schrag D. (2017). Overall survival results of a trial assessing patient-reported outcomes for symptom monitoring during routine cancer treatment. JAMA.

[4] Basch E., Stover A.M., Schrag D., Chung A., Jansen J., Henson S., Carr P., Ginos B., Deal A., Spears P.A. (2020). Clinical utility and user perceptions of a digital system for electronic patient-reported symptom monitoring during routine cancer care: Findings from the PRO-TECT trial. JCO Clin. Cancer Inform..

[5] Jauhari Y., Gannon M.R., Dodwell D., Horgan K., Tsang C., Clements K., Medina J., Tang S., Pettengell R., Cromwell D.A. (2020). Addressing frailty in patients with breast cancer: A review of the literature. Eur. J. Surg. Oncol..

[6] Warrington L., Absolom K., Conner M., Kellar I., Clayton B., Ayres M., Velikova G. (2019). Electronic systems for patients to report and manage side effects of cancer treatment: Systematic review. J. Med. Internet Res..

[7] Kamgar M., Greenwald R.K., Asad H., Hastert T.A., McLaughlin E.M., Reding K.W., Paskett E.D., Bea J.W., Shadyab A.H., Neuhouser M.L. (2021). Prevalence and predictors of peripheral neuropathy after breast cancer treatment. Cancer Med..

[8] Bandos H., Melnikow J., Rivera D.R., Swain S.M., Sturtz K., Fehrenbacher L., Wade J.L., Brufsky A.M., Julian T.B., Margolese R.G. (2017). Long-term peripheral neuropathy in breast cancer patients treated with adjuvant chemotherapy: NRG Oncology/NSABP B-30. J. Natl. Cancer Inst..

[9] Zhi W.I., Baser R.E., Kwon A., Chen C., Li S.Q., Piulson L., Seluzicki C., Panageas K.S., Harte S.E., Mao J.J. (2021). Characterization of chemotherapy-induced peripheral neuropathy using patient reported outcomes and quantitative sensory testing. Breast Cancer Res. Treat..

[10] National Cancer Institute (2023). Patient-Reported Outcomes Version of the Common Terminology Criteria for Adverse Events (PRO-CTCAE^®^). https://healthcaredelivery.cancer.gov/pro-ctcae/.

[11] Fayers P., Bottomley A. (2002). EORTC Quality of Life Group; Quality of Life Unit; European Organisation for Research and Treatment of Cancer. Quality of life research within the EORTC—The EORTC QLQ-C30. Eur. J. Cancer.

[12] Jensen R.E., Potosky A.L., Reeve B.B., Hahn E., Cella D., Fries J., Smith A.W., Keegan T.H., Wu X.C., Paddock L. (2015). Validation of the PROMIS physical function measures in a diverse US population-based cohort of cancer patients. Qual. Life Res..

[13] Pearman T.P., Beaumont J.L., Mroczek D., O’Connor M., Cella D. (2018). Validity and usefulness of a single-item measure of patient-reported bother from side effects of cancer therapy. Cancer.

[14] Basch E., Becker C., Rogak L.J., Schrag D., Reeve B.B., Spears P., Smith M.L., Gounder M.M., Mahoney M.R., Schwartz G.K. (2021). Composite grading algorithm for the National Cancer Institute’s Patient Reported Outcomes version of the Common Terminology Criteria for Adverse Events (PRO-CTCAE). Clin. Trials.

[15] Palumbo A., Bringhen S., Mateos M.V., Larocca A., Facon T., Kumar S.K., Offidani M., McCarthy P., Evangelista A., Lonial S. (2015). Geriatric assessment predicts survival and toxicities in elderly myeloma patients: An International Myeloma Working Group report. Blood.

[16] Katz J.N., Chang L.C., Sangha O., Fossel A.H., Bates D.W. (1996). Can comorbidity be measured by questionnaire rather than medical record review?. Med. Care.

[17] Hurria A., Gupta S., Zauderer M., Zuckerman E.L., Cohen H.J., Muss H., Rodin M., Panageas K.S., Holland J.C., Saltz L. (2005). Developing a cancer-specific geriatric assessment. Cancer.

[18] Rockwood K., Mitnitski A. (2007). Frailty in relation to the accumulation of deficits. J. Gerontol. A Biol. Sci. Med. Sci..

[19] Searle S.D., Mitnitski A., Gahbauer E.A., Gill T.M., Rockwood K. (2008). A standard procedure for creating a frailty index. BMC Geriatr..

[20] Oken M.M., Creech R.H., Tormey D.C., Horton J., Davis T.E., McFadden E.T., Carbone P.P. (1982). Toxicity and response criteria of the eastern cooperative oncology group. Am. J. Clin. Oncol..

[21] Martin L., Watanabe S., Fainsinger R., Lau F., Ghosh S., Quan H., Atkins M., Fassbender K., Downing G.M., Baracos V. (2010). Prognostic factors in patients with advanced cancer: Use of the patient-generated subjective global assessment in survival prediction. J. Clin. Oncol..

[22] Basch E., Hudson K., Rocque G. (2023). Implementation of electronic patient-reported outcomes for symptom monitoring during cancer treatment: The importance of getting it right. J. Comp. Eff. Res..

[23] Alhazmi L.S.S., Bawadood M.A.A., Aljohani A.M.S., Alzahrani A.A.R., Moshref L., Trabulsi N., Moshref R. (2021). Pain management in breast cancer patients: A multidisciplinary approach. Cureus.

[24] So W.K.W., Law B.M.H., Ng M.S.N., He X., Chan D.N.S., Chan C.W.H., McCarthy A.L. (2021). Symptom clusters experienced by breast cancer patients at various treatment stages: A systematic review. Cancer Med..

[25] Pereira S., Fontes F., Sonin T., Dias T., Fragoso M., Castro-Lopes J., Lunet N. (2017). Neuropathic pain after breast cancer treatment: Characterization and risk factors. J. Pain Sym. Manag..

